# Supplementary material for: Impact of Sarcopenia on Clinical Outcomes in a Cohort of Caucasian Active Crohn’s Disease Patients Undergoing Multidetector CT-Enterography
Source: Nutrients. 2022 Aug 23;14(17):3460. doi: 10.3390/nu14173460 (PMC9458031; doi:10.3390/nu14173460)
Supplement: Supplementary file 1 [file nutrients-14-03460-s001.zip › nutrients-1860505-supplementary.pdf]

Supplementary Table S1. Calculated probability of being sarcopenic based on increasing values of BMI and on the presence of extraintestinal manifestations (EIMs). 0, absence of EIMs; 1, presence of EIMs.

BMI (kg/m²)	EIM		BMI (kg/m²)	EIM		BMI (kg/m²)	EIM	
	0	1		0	1		0	1
15	0.902	0.994	20.0	0.663	0.974	25.0	0.295	0.890
15.1	0.899	0.994	20.1	0.656	0.973	25.1	0.289	0.887
15.2	0.896	0.994	20.2	0.649	0.973	25.2	0.283	0.883
15.3	0.894	0.994	20.3	0.642	0.972	25.3	0.276	0.880
15.4	0.891	0.994	20.4	0.634	0.971	25.4	0.270	0.877
15.5	0.888	0.993	20.5	0.627	0.970	25.5	0.264	0.874
15.6	0.884	0.993	20.6	0.620	0.969	25.6	0.258	0.870
15.7	0.881	0.993	20.7	0.613	0.968	25.7	0.252	0.867
15.8	0.878	0.993	20.8	0.605	0.967	25.8	0.247	0.863
15.9	0.875	0.993	20.9	0.598	0.966	25.9	0.241	0.859
16.0	0.871	0.992	21.0	0.590	0.965	26.0	0.235	0.855
16.1	0.868	0.992	21.1	0.583	0.964	26.1	0.230	0.852
16.2	0.864	0.992	21.2	0.575	0.963	26.2	0.224	0.848
16.3	0.860	0.992	21.3	0.568	0.962	26.3	0.219	0.844
16.4	0.857	0.991	21.4	0.560	0.961	26.4	0.214	0.839
16.5	0.853	0.991	21.5	0.553	0.960	26.5	0.209	0.835
16.6	0.849	0.991	21.6	0.545	0.958	26.6	0.204	0.831
16.7	0.845	0.991	21.7	0.537	0.957	26.7	0.199	0.827
16.8	0.841	0.990	21.8	0.530	0.956	26.8	0.194	0.822
16.9	0.837	0.990	21.9	0.522	0.955	26.9	0.189	0.818
17.0	0.832	0.990	22.0	0.514	0.953	27.0	0.184	0.813
17.1	0.828	0.989	22.1	0.507	0.952	27.1	0.180	0.808
17.2	0.823	0.989	22.2	0.499	0.950	27.2	0.175	0.803
17.3	0.819	0.989	22.3	0.491	0.949	27.3	0.171	0.798
17.4	0.814	0.988	22.4	0.483	0.947	27.4	0.166	0.793
17.5	0.810	0.988	22.5	0.476	0.946	27.5	0.162	0.788
17.6	0.805	0.988	22.6	0.468	0.944	27.6	0.158	0.783
17.7	0.800	0.987	22.7	0.460	0.943	27.7	0.154	0.778
17.8	0.795	0.987	22.8	0.453	0.941	27.8	0.150	0.772
17.9	0.790	0.986	22.9	0.445	0.939	27.9	0.146	0.767
18.0	0.785	0.986	23.0	0.437	0.937	28.0	0.142	0.761
18.1	0.779	0.986	23.1	0.430	0.935	28.1	0.138	0.756
18.2	0.774	0.985	23.2	0.422	0.934	28.2	0.135	0.750
18.3	0.769	0.985	23.3	0.415	0.932	28.3	0.131	0.744
18.4	0.763	0.984	23.4	0.407	0.930	28.4	0.128	0.738
18.5	0.757	0.984	23.5	0.400	0.928	28.5	0.124	0.732
18.6	0.752	0.983	23.6	0.392	0.926	28.6	0.121	0.726

18.7	0.746	0.983	23.7	0.385	0.923	28.7	0.118	0.720
18.8	0.740	0.982	23.8	0.378	0.921	28.8	0.115	0.714
18.9	0.734	0.982	23.9	0.370	0.919	28.9	0.112	0.707
19.0	0.728	0.981	24.0	0.363	0.917	29.0	0.109	0.701
19.1	0.722	0.980	24.1	0.356	0.914	29.1	0.106	0.694
19.2	0.715	0.980	24.2	0.349	0.912	29.2	0.103	0.688
19.3	0.709	0.979	24.3	0.342	0.909	29.3	0.100	0.681
19.4	0.703	0.978	24.4	0.335	0.907	29.4	0.097	0.674
19.5	0.696	0.978	24.5	0.328	0.904	29.5	0.094	0.667
19.6	0.690	0.977	24.6	0.322	0.901	29.6	0.092	0.661
19.7	0.683	0.976	24.7	0.315	0.898	29.7	0.089	0.654
19.8	0.676	0.976	24.8	0.308	0.896	29.8	0.087	0.647
19.9	0.669	0.975	24.9	0.302	0.893	29.9	0.084	0.639
